# From polyethylene waste bottles to UIO-66 (Zr) for preconcentration of steroid hormones from river water

**DOI:** 10.1038/s41598-023-34031-z

**Published:** 2023-04-26

**Authors:** Shirley Kholofelo Selahle, Azile Nqombolo, Philiswa Nosizo Nomngongo

**Affiliations:** 1 Department of Chemical Sciences, University of Johannesburg, Doornfontein Campus, P.O. Box 17011, Doornfontein, 2028 South Africa; 2 Department of Science and Innovation-National Research Foundation South African Research Chair Initiative (DSI-NRF SARChI): Nanotechnology for Water, University of Johannesburg, Doornfontein, 2028 South Africa; 3 Department of Pure and Applied Chemistry, University of Fort Hare, Alice, 5700 South Africa

**Keywords:** Environmental sciences, Chemistry

## Abstract

Metal–organic framework (UiO-66 (Zr) was synthesized using polyethylene terephthalate (PET) and used as an adsorbent for extraction and preconcentration of steroid hormones in river water. Polyethylene waste bottles were used as the source of polyethylene terephthalate (PET) ligands. The UIO-66(Zr), which the PET was made from recycled waste plastics, was used for the first time for the extraction and preconcentration of four different types of steroid hormones in river water samples. Various analytical characterization techniques were employed to characterize the synthesized material. The steroid hormones were detected and quantified using high-performance liquid chromatography coupled with diode array detector (HPLC–DAD). The results were further validated using ultra-high performance liquid chromatography-tandem mass spectrometry (UHPLC-MS/MS). Experimental variables, such as sample pH, the mass of adsorbent and extraction time, were optimized using Box-Behnken design (BBD). The dispersive solid phase extraction method combined with HPLC–DAD, displayed good linearity (0.004–1000 µg/L) low limits of detections (LODs, 1.1–16 ng/L for ultrapure water and 2.6–5.3 ng/L for river water) and limits of quantification (LOQs, 3.7–5.3 ng/L for ultrapure water and 8.7–11.0 ng/L for river water samples) and acceptable extraction recoveries (86–101%). The intraday (n = 10) and interday (n = 5) precisions expressed in terms of relative standard deviations (%RSD) were all less than 5%. The steroid hormones were detected in most of the river water samples (Vaal River and Rietspruit River). The DSPE/HPLC method offered a promising approach for simultaneous extraction, preconcentration and determination of steroid hormones in water.

## Introduction

Steroid hormones are known as active chemical compounds that are involved in all the significant physiological roles in the body of living organisms^1^. These roles include the development of sexual characteristics, regulation of cell activity, mood control, water balance in the body, stimulation of the metabolism activities and growth, among others^2–4^. Steroid hormones are classified into four groups based on their structural variation and affinities^5^. These groups include oestrogens, androgens, progestogens, and corticosteroid^6^. Oestrogen and progestogens are taken for hormones replacement in the body, orally and non-oral contraceptives^7^. Corticosteroids are used in humans for several pathologies such as malignant tumours, skin diseases and rheumatism^8^. These hormones are widely used worldwide by both humans and animals, and subsequently reach water systems through excretion, direct discharge of wastewater to the water sources and wastewater treatment plants (WWTPs) effluents^9^. This could be a serious concern given that conventional water treatment techniques have proven to be inefficient to appropriately remove emerging organic pollutants^10^. Although steroid hormones are present in environmental water systems at low concentrations, they can act as endocrine disrupting compounds, causing adverse effect to humans, animals, and aquatic life^11^. Therefore, it is necessary to develop analytical methods that will enable simultaneous analysis of steroid hormones and provide a comprehensive understanding of steroid hormone contamination in water systems.

To date, various analytical techniques have been employed for the detection, separation, and quantification of steroid hormones in water matrices. These analytes are frequently detected and quantified using high performance liquid chromatography- diode array detector (HPLC–DAD)^12^, HPLC- fluorescence detector (HPLC-FLD)^13^, liquid chromatography coupled with tandem mass spectrometry (LC–MS/MS)^14^, gas chromatography with tandem mass spectrometry (GC–MS/MS)^15^, capillary electrophoresis (CE)^16^, amongst others. Despite great advancements in analytical instrumentation, direct analysis of the sample without sample preparation step its difficult^17^.This is due to the low concentration levels of steroid hormones in water systems, as well as the complexity of the sample matrices. Therefore, various sample pre-treatment methods are often coupled with analytical detection techniques for sample clean up, extraction and preconcentration of analytes of interest^18^. Sample preparation becomes a very important step in environmental analysis as it improves selectivity and sensitivity of the analytical instrument^17^.

As a result, different sample preparation methods have been developed and utilized for the analysis of steroid hormones in water matrices. These sample treatment methods include dispersive liquid–liquid microextraction (DLLME)^19^, hollow fibre liquid–liquid microextraction (HF-LLME)^20^, dispersive solid phase extraction (DSPE)^21^, magnetic solid phase extraction (MSPE)^22^, traditional solid phase extraction (SPE)^23^, solid phase microextraction (SPME)^24^ and stir-bar sorptive extraction (SBSE)^25^, amongst others. Sorbent base sample preparation methods have been commonly used for the extraction and preconcentration of steroid hormones in water samples. This is because they offer crucial benefits such as enrichment of trace analytes from complex matrices, sample matrix elimination, minimal chances of cross-contamination effective extraction with satisfactory percentage recoveries, and use of different adsorbents^26^. However, traditional SPE presents some disadvantages such as the need to purchase expensive sorbents and the blockage of cartridges^27^. Therefore, due to of the conventional SPE drawback, DSPE was later introduced^28^. DSPE is known to offer advantages such as being easy, rapid and inexpensive technique that can be used to clean up, extract and preconcentrate different analytes in real samples with high enrichment factor, high extraction efficiency, and it requires low volume of the sample^29^.

Similar to traditional SPE, a choice of an adsorbent plays a crucial part in DSPE^30^. Therefore, various adsorbent materials with high adsorption capacity, improved stability, and enhanced extraction efficiency, as well as high affinity and selectivity towards the target analytes have been reported in literature. These include amino-functionalized metal–organic frameworks^31^, Magnetic GO/γ-Fe_2_O_3_ nanoparticles^13^, sodium dodecyl sulfate- multi-walled carbon nanotubes^32^, functional groups-rich graphene oxide and carbon nanotubes^22^ and Fe_3_O_4_-Al_2_O_3_@CNFs^21^. Among the above mentioned adsorbent materials, metal–organic frameworks (MOFs) have been receiving increased consideration as sorbents in DSPE. MOFs are prepared by a simple procedure of self-assembly of organic linkers and metal ions via coordination bonds^33^. Owing to their chemical and thermal stability, high surface area, porous structure and uniformly structured cavities^34^. MOFs have been widely used in sample pre-treatment for analysis of various organic contaminants such as pesticides^35^, pharmaceuticals^36^, personal care products^36^, food colourants^37^ and various trace elements^38^. Among numerous MOFs, UiO-66 displays outstanding features such as stability, often exhibit high specific surface areas, controllable porosity, tuneable pore walls, strong affinity towards analytes and flexible chemical composition due to the presence of strong chemical bonds and adaptable organic linking units^39^.

Plastic pollution in the world become an ecological disaster of the twenty-first century, which deserves a serious attention^40^. In 2015, about 6400 million tons of plastics were made, and only 9% was recycled, 12% was burnt, 79% was accumulated in landfills, and if these trends continue, roughly 12,000 million tons will be contaminating the environment^40^. Polyethylene terephthalate (PET) is one of the most produced and used polymers in the world, which contributed to the contamination largely by the packaging industry^41^. To minimize pollution, plastic wastes were recycled for making PET ligands for UIO-66(Zr).

In this study PET based UIO-66(Zr) MOF has been used as a sorbent for simultaneous extraction and preconcentration of four steroid hormones from river water samples. Plastic waste was recycled to prepare PET which was used as a ligand in the formation of PET based UIO-66(Zr). Making use of PET based UIO-66(Zr) MOF for adsorption is safe to the environment rather than using terephthalic acid used in other UIO-66(Zr) which has been reported to leach in environment and end up being toxic to living organisms^42,43^. On the other hand, making use of the plastic waste for making adsorbents will assist in reducing plastic waste disposed all over the country^44^.The analytes in the samples were quantified by high performance liquid chromatography coupled to a diode array detector and liquid chromatography coupled with mass spectrometry (LC–MS). According to literature there are few or no related studies where PET based UIO-66(Zr) was used as a sorbent for extraction and preconcentration of steroid hormones in water samples^45,46^. The experimental influential parameters were optimized by Box Behnken design, which offers advantages of finding out the possible interactions between experimental parameters and timesaving by reducing the number of experiments to be carried out. Lastly, the designed method was then validated and applied for quantifying and analysing the targeted steroid hormones in river water samples. To the best of our knowledge, this is the first in literature where a PET based UIO-66(Zr) was used for the simultaneous extraction and preconcentration of steroid hormones in river water samples. The PET used in the synthesis of the UIO-66(Zr) was made from waste plastic. This MOF indicated its effectiveness by being able to extract four types of steroid hormones simultaneously. However, various types of UIO-66(Zr) have been reported for the extraction of various organic pollutants.

## Results and discussions

### Characterization of PET based UIO-66(Zr)

The functional groups of PET based UIO-66(Zr) were examined and confirmed using FTIR (Fig. S1). The strong adsorption band at 3394 cm^−1^ and vibration peak of 1661 cm^−1^ were assigned to the hydroxyl (–OH) and carbonyl (–C=O) groups of the carboxylic acid of the ligand in PET based UIO-66(Zr) material, respectively. The adsorption band at 1576 cm^−1^ was ascribed to C=C bond of the benzene ring of the PET ligand and the vibration band at wavenumbers 1391 cm^−1^ and 1096 cm^−1^ confirmed the presence of a C–O and C–O–C stretching vibration for the carboxylic acid group. In addition, the peaks at 730–478 cm^−1^ were assigned Zr–O confirming the coordination of Zr metal centres in PET based UIO-66(Zr) to the oxygen atom of the ligand. These findings were similar to the ones reported by^47,48^, even though they made use of commercial ligands such as terephthalic acids.

**Figure 1:**
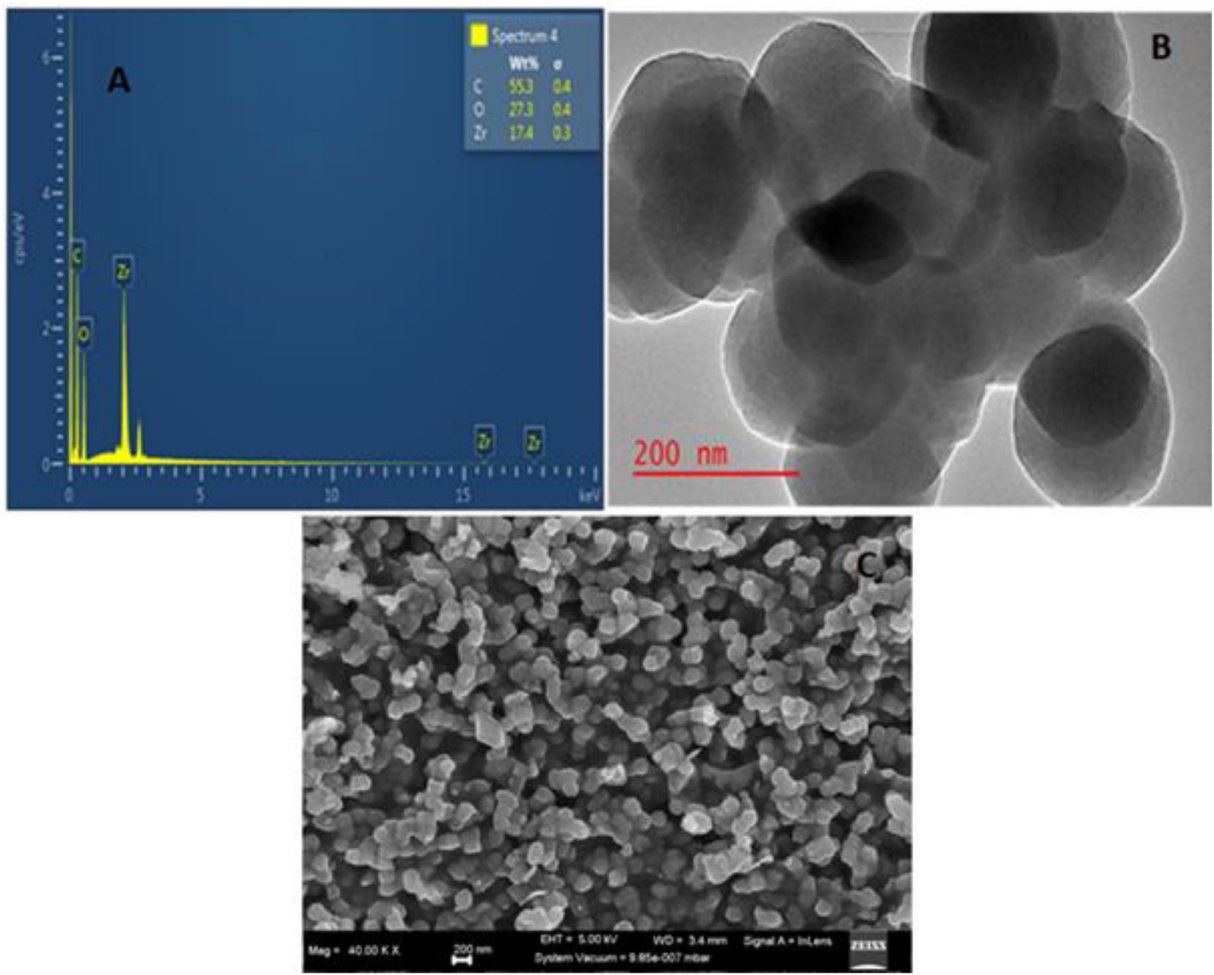
(**A**) EDS, (**B**) TEM and (**C**) HR-SEM image of PET based UIO-66(Zr).

The EDS image (Fig. 1a) showed that all the expected elements, carbon, oxygen, and zirconium were found in the prepared PET based UIO-66(Zr). The TEM image Fig. 1b indicated that PET based UIO-66(Zr) was composed of spherical particles. which were uniform in size. HR-SEM depicted in Fig. 1c further confirmed that PET based UIO-66(Zr) was indeed composed of spherical particles. These findings were similar to the one reported by^49^. The EDS pattern of PET based UIO-66(Zr) is shown in Fig. 1a, with carbon (C) and oxygen (O) from the ligand then zirconium (Zr) from the metal salt. The introduction of the PET obtained from recycled plastic waste intermingled well with the UIO-66 since it did not show any foreign peaks.

**Figure 2:**
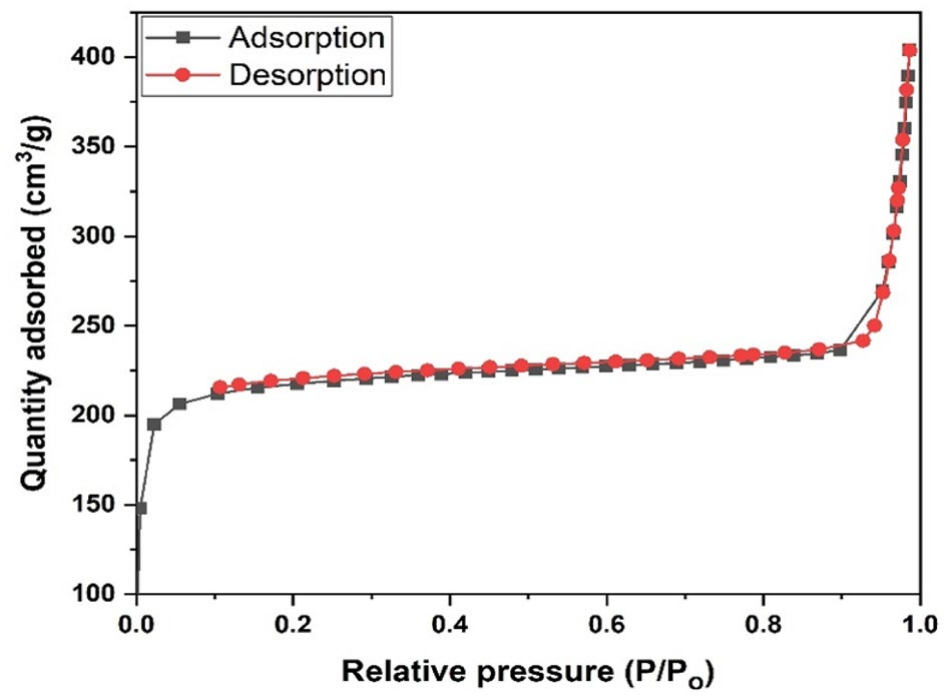
N_2_ adsorption/desorption isotherms for PET based UIO-66(Zr).

The N_2_ adsorption/desorption isotherms were used to examine the surface properties of the prepared PET based UIO-66(Zr) (Fig. 2). The surface area of the was found to be 1311 m^2^ g^−1^ with a pore volume and an average pore size of 0.62 m^3^ g^−1^ and 3.76 nm, respectively. Figure 2 indicates the N_2_ adsorption/desorption isotherms plots for PET based UIO-66(Zr) indicated a type IV isotherms and the tiny H_3_ hysteresis loop and the pore size showed that the PET based UIO-66(Zr) was mesoporous in nature^50^.

### Point of zero charge

Point of zero charge (pH_pzc_) experiments were carried out to examine the pH which positive and negative charges have the same magnitude on the surface of PET based UIO-66(Zr). This concept gives an insight about the surface science of the synthesized PET based UIO-66(Zr). The change in pH results were plotted against the pH of the aqueous solutions, in the range of pH 2 until pH 12 as shown from Fig. S2. The surface charge of PET based UIO-66(Zr) was found to be positively charged from low pH values below pH 8 and negatively charge above pH 8. Furthermore, the pH at point of zero charge of PET based UIO-66(Zr) composite was 8 according to the plot.

### Selection of eluent type

The elution conditions play a vital role in the desorption of steroid hormones from the adsorbent because they can significantly affect the performance sample pre-treatment method. In the study, the capabilities of five solvents (including combination of solvents) were investigated for effective elution of hormones from the PET based UIO-66(Zr) material. The suitable solvents for desorption of analytes from the adsorbent is often dictated by the solubility of the analytes and the relative solvent polarity^51^. The polarity of pure solvents was in the following order: acetonitrile (ACN) > methanol (MeOH) > ethanol (EtOH). Therefore, the polarity of five desorption solvents was as follows: ACN > MeOH: ACN (1:1) > MeOH > EtOH: EtOH (1:1) > EtOH. Figure S3 revealed that the significantly higher extraction recoveries (%R) of all steroid hormones were obtained when methanol was used as the eluent compared to other solvent used.

This phenomenon was attributed to the structural difference between the solvents and polarity. In this case, MeOH could easily enter the PET based UIO-66(Zr) pore structures and replace the analytes of interest, thus attaining complete elution^52^. As seen in Fig. S3, ACN was also suitable for progesterone. This is because this progesterone does not have hydroxyl (-OH) groups in the skeleton structure^53^. These findings demonstrate that the stronger polar solvent (ACN) was also more favourable for the elution of polar target progesterone. For simplicity, methanol was selected for consequent experiments.

### Optimization strategy

The effects of the independent variables namely, mass of sorbent (MA), extraction time (ET), pH and eluent volume (EV) on the extraction and preconcentration of steroid hormones were investigated using RSM. The BBD matrix was composed of 27 experimental runs, performed in triplicates. The respective analytical response (percentage extraction recoveries (%R) are presented in Table S1 in the supplementary data. A TIBCO® Statistica™ package version 13 (StatSoft, Palo Alto, CA, USA) was used to process the data. The analysis of variance (ANOVA) was used to examine the importance of independent variables and their interactions on the analytical response. These results suggested that there was a good relationship between experimental data and predicted values. Therefore, the RSM model can be used for satisfactory response prediction and is suitable for optimization of the experimental variables. The ANOVA findings were illustrated in the form of Pareto Chart shown in Fig. S4. These charts revealed that the sample pH was significant for all the analytes (Fig. S4a–d). In contrast, Fig. S4b shows that the mass of sorbent and the sample pH and were significant at the 95% confidence level.

#### Response surface methodology

Response surface methodology was carried out to investigate the interactive effects of independent variables. Figure S5A–F presents the interaction within sample pH, mass of adsorbent, extraction time and eluent volume. Figure S5 revealed that sample pH played an important role in the extraction of the analytes. This is because this parameter influences the surface charge of the adsorbent and the chemical species of the analytes. Interactive effects of MA and pH on the %R (while other parameters are fixed at their central points) are presented in Fig. S5A. On the other hand, Fig. S5B,C show the simultaneous effect of pH combined with ET and EV on the analytical response. As seen in Fig. S5A–C, the combined effects of these parameters are very significant, and this is visualised by relatively steep response surface curve.

According to Table S2, the pKa values for estrone, 17β-estradiol and hydrocortisone and progesterone were 10.25, 10.27, 12.58. It should be noted that there is no pKa value reported for progesterone. The results in Fig. S5A–C suggest that pH had more influence as compared to other parameters. This is because at pH values lower that the pKa values of the target analytes, the neutral forms of the steroid hormones were dominant in the solution. This suggested that the adsorption mechanism could be driven by π–π electron donor–acceptor interactions and hydrogen bonding. On the other hand, the analytical response reached the maximum between pH 5 and 7. The pH_pzc_ of the adsorbent was found to be 8.0 (Fig. S2). This suggest that below the pH_pzc_ value, electrostatic interaction between the electron rich analytes and positively charged adsorbent occurs, thus leading to quantitative analytical response. The response surface plot in Fig. S5D displays relatively circular shape suggesting that the interactions between ET and EV were not significant. This is expected because these parameters are not related, ET is responsible for the adsorption and interaction between the analytes and adsorbent while EV is responsible for desorption of analytes from the solid material. Figure S5E shows that quantities extraction of analytes could be achieved at higher MA (> 18 mg) and when ET is above 26 min. This is because more adsorption sites are available for adsorption of the analytes at higher MA. In addition, these findings suggest that more time is required to reach maximum interaction between MA and analytes. The interactive effect between MA and EV was not significant, suggesting that the eluent was strong enough to reverse the interaction between the adsorbent and the analytes.

#### Determination of optimum conditions using desirability function

**Figure 3:**
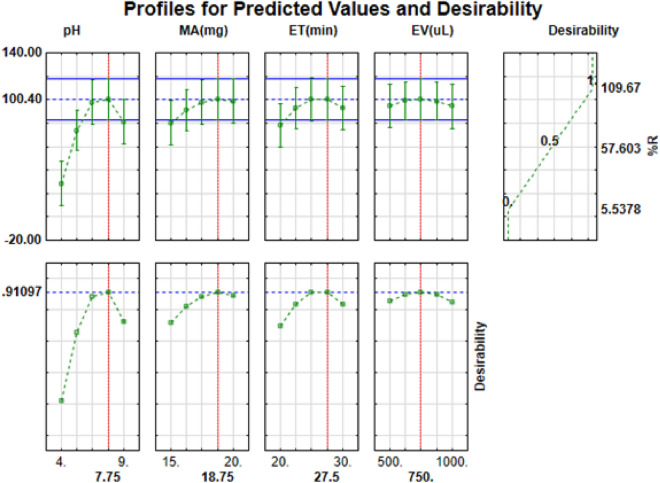
Desirability function for optimization of independent parameters for the targeted steroid hormones. A TIBCO® Statistica™ package version 13 (StatSoft, Palo Alto, CA, USA) (https://edelivery.tibco.com/storefront/eval/tibco-statistica-desktop/prod11850.html).

Figure 3 shows the desirability profiles for the estimation of experimental optimum conditions. Figure 3 illustrates the desirable percentage recoveries as well as optimum conditions for extraction and preconcentration of the hormones. The desired percentage recoveries predicated by the plots in the model are presented in the top left-hand side plot. The experimental percentage recoveries related to desirability values of 0.0 (least), 0.5 (central) and 1.0 (maximum) are presented in top right-hand side plot. To achieve maximum recoveries of the analytes, the desirability score of 1.0 (maximum, bottom left-hand side plot) was selected as the target value for the optimization of the influential parameters. As shown in Fig. 3, desirability score of 1.0 resulted to maximum percentage recovery of 100.4% when the optimum conditions were 7.7, 19 mg, 27.5 min and 750 µL for pH, mass of adsorbent extraction time and eluent volume, respectively. To verify and validate the predicted results, the extraction and preconcentration of target analytes was carried out experimentally using the estimated optimum conditions. The experimental results obtained are presented in Fig. 3. As seen, experimental %R agreed with the predicted values. This exhibited that RSM model based on BBD was effective for optimization of the developed DSPE method.

### Adsorption capacities

The adsorption capacities of PET based UIO-66(Zr) For the adsorption of steroid hormones were examined under optimum conditions. To investigate the adsorption capacities of the steroid hormones, 100 mg/L of hormones mixture was prepared in ultrapure water and sonicated for a period of 28 min at room temperature (25 °C). Concentrations found at equilibrium systems were analyzed using the HPLC–DAD and the obtained adsorption capacities for the individual analytes are shown in Table S5. The adsorption capacities of the target analytes were calculated using Eq. (1).

1 $${q}_{e}=\left(\frac{{C}_{0}-{C}_{e}}{m}\right)V$$

where q_e_ is the adsorption capacity, C_0_ is the initial concentration, C_e_ is the equilibrium concentration, m represent mass of sorbent and V represents the sample volume.

### Analytical performance

The analytical figures of merit were evaluated by investigating the precision (intraday and inter-day), accuracy (or trueness), matrix effect, specificity, linearity, calibration curves, coefficient of determination, limits of quantification (LOQs) and limits of detection (LODs) are shown in Tables 1 and 2.

**Table 1:** Analytical characteristics of the developed method.

	17β-estradiol	Estrone	Hydrocortisone	Progesterone
Regression equation	y = 1.431x + 0.173	y = 1.246x + 0.205	y = 1.912x + 0.384	y = 1.283x + 0.267
	y = 1.343x + 0.562	y = 1.186x + 0.347	y = 1.713x + 0.615	y = 1.178x + 0.452
R^2^	0.9989	0.9991	0.9996	0.9997
	0.9981	0.9976	0.9986	0.9984
Linearity (µg/L)	0.004–750	0.005–950	0.0055–1000	0.0045–850
	0.011–800	0.011–800	0.009–950	0.009–950
Matrix effect (%ME)	− 6.1	− 4.8	− 10.4	− 8.2
Enhancement factor	83.7	61.1	49.9	49.2

**Table 2:** Limits of detection and quantification from ultrapure water and river water samples.

Analytes	LOD (ng/L)		LOQ (ng/L)		Intraday (%RSD)^a^			Interday (%RSD)^b^		
	Ultrapure water	River water	Ultrapure water	River water	5.0	50	100	5.0	50	100
17β-estradiol	1.1	3.2	3.7	10.7	4.5	3.6	2.4	5.3	4.6	3.2
Estrone	1.5	3.3	5.0	11.0	4.1	3.1	3.5	5.4	3.7	2.7
Hydrocortisone	1.6	2.6	5.3	8.7	4.7	4.3	3.9	5.6	3.5	2.6
Progesterone	1.4	2.7	4.7	9.0	4.3	4.6	2.5	4.9	3.3	2.8

^a^Same day measurements (intraday) n = 10.

^b^Measurement done at different days (interday), n = 5 (each day analysis was done in triplicates).

#### Calibration curves, linearity, and matrix effect

The suppression or enhancement of the signal is often produced by co-existing interferences that are co-eluted with the target analytes. To evaluate the matrix effects, the extracts of matrix matched, and pure standard solutions were compared. The %ME ranged between − 2.3 and − 8.9 indicating that there was minimal or no apparent response suppression or enhancement after the samples were pre-treated with DSPE as shown in Table 1.

#### Limits of detection and quantification

See Table 2.

#### Accuracy, precision, and specificity

The accuracy of the developed method was determined using spiked recovery experiments. To achieved this by spiking two river water samples at three concentration levels that is, low (5.0 ng/L), medium (10 ng/L), high concentrations (100 ng/L). The results in Table 3 and Fig. S6 reveals that there was a good agreement between the added and obtained concentrations with recoveries ranging 87–101% with RSDs of 1.8–4.1%.

**Table 3:** Determination of steroid hormones in spiked river was samples. (average ± std deviation).

Samples	Added	17β-estradiol			Estrone			Hydrocortisone			Progesterone		
		Found	%R	%RSD	Found	%R	%RSD	Found	%R	%RSD	Found	%R	%RSD
RRU S1^a^	0	74.9 ± 1.6	–	2.1	87.5 ± 2.1	–	2.4	30.1 ± 0.9	–	3.1	12.5 ± 0.5	–	3.7
	5.0	79.7 ± 1.5	96.7	1.9	92.4 ± 1.8	97.3	1.9	34.9 ± 0.9	96.6	2.6	17.2 ± 0.6	94.6	3.4
	50	125 ± 3	99.2	2.2	137 ± 4	98.7	2.9	79.7 ± 2.1	99.1	2.5	61.5 ± 1.8	97.9	2.9
	100	174 ± 3	99.1	1.8	187 ± 3	99.5	1.6	129 ± 5	99.1	3.9	112 ± 2	99.5	1.8
VRB S8^b^	0	< LOQ	–	–	< LOQ	–	–	10.9 ± 0.4	–	4.1	13.8 ± 0.5	–	3.5
	5.0	4.34 ± 0.15	86.7	3.5	4.42 ± 0.20	88.4	3.7	15.2 ± 0.6	94.5	3.8	18.6 ± 0.6	95.3	3.1
	50	48.2 ± 1.1	96.3	2.3	48.8 ± 1.1	97.5	2.3	60.0 ± 2.2	98.9	3.6	63.3 ± 1.6	99.0	2.6
	100	98.7 ± 2.1	98.7	2.1	98.1 ± 2.5	98.1	2.5	110 ± 4	99.4	3.6	115 ± 3	101	2.6

^a^RRU SI: Rietspruit River upstream (before the wastewater treatment plant (WWTP).

^b^VRB S8: Vaal River before Rietspruit River joins.

Table S6 displays the analytical performance of the reported SPE methods from literature with the designed DSPE-HPLC–DAD method. The developed method showed to have better sensitivity in terms of the linearity and the LODs as compared to^21,54^ and^22^. However,^22,55^ reported more better LODs and linearity than the developed method. This was attributed to the fact that they used more advanced analytical instruments such as UHPLC and HPLC MS/MS for their detection.

### Application to real samples

The applicability of the developed method was investigated by analysing river waters samples. The concentrations detected are shown in Table 4 and Fig. S7 shows chromatograms of Rietspruit river water after extraction using the developed method. The chromatograms are smooth and free from interferences from the complex matrix, showing the designed method was able to preconcentrate and extract different steroid hormones from river water. To further examine the accuracy of the developed DSPE/HPLC–DAD method, confirmatory experiments with reference method (DSPE/LC–MS) were performed. It can be seen from Table 4 that the concentrations of steroid hormones detected with DSPE/HPLC–DAD method agreed with those obtained using DSPE/LC–MS. These results are evidence that the developed method has acceptable accuracy and precision.

**Table 4:** Application to real water samples, n = 3 (average ± std deviation).

Samples	Concentrations (ng/L)							
	DSPE/HPLC–DAD	DSPE/LC–MS	DSPE/HPLC–DAD	DSPE/LC–MS	DSPE/HPLC–DAD	DSPE/LC–MS	DSPE/HPLC–DAD	DSPE/LC–MS
	Estrone		17β-estradiol		Hydrocortisone		Progesterone	
RRU S2	135 ± 5	133 ± 5	174 ± 4	171 ± 4	23.1 ± 0.5	23.5 ± 0.3	15.6 ± 0.3	16.1 ± 0.5
RRU S3	105 ± 3	109 ± 3	211 ± 9	208 ± 5	108 ± 3	110 ± 4	10.5 ± 0.4	11.1 ± 0.5
RRD S4	178 ± 5	182 ± 4	196 ± 7	201 ± 4	128 ± 4	131 ± 3	16.8 ± 0.5	16.3 ± 0.9
RRD S5	223 ± 6	225 ± 7	781 ± 10	778 ± 8	78.3 ± 0.4	77.2 ±	22.1 ± 0.6	21.7 ± 0.7
RRD S6	318 ± 7	314 ± 8	613 ± 9	609 ± 6	113 ± 3	111 ± 2	25.6 ± 0.3	26.1 ± 0.5
RRD S7	189 ± 4	191 ± 4	432 ± 6	435 ± 5	67.8 ± 0.3	68.4 ± 0.7	12.7 ± 0.9	13.2 ± 0.8
VRB S9	14.5 ± 0.7	15.3 ± 0.3	77.7 ± 0.8	81.3 ± 0.4	ND	ND	ND	ND
VRA S10	77.3 ± 0.4	76.8 ± 0.9	127 ± 4	130 ± 4	54.6 ± 0.5	55.1 ± 0.6	ND	ND
VRD S11	15.5 ± 0.3	16.3 ± 0.5	25.6 ± 0.3	24.8 ± 0.3	34.5 ± 0.3	35.6 ± 0.7	ND	ND
VRD 12	6.78 ± 0.05	6.83 ± 0.04	9.55 ± 0.05	9.67 ± 0.04	ND	ND	ND	ND

The detection of steroid hormones in the water systems has been reported to differ from country-to-country as shown in Table S7. The steroid hormone concentrations detected in this study were compared to other globally reported concentrations. The obtained concentrations were found to be above than the other countries reported in Table S7, However, Brazil reported a concentration which had a similar magnitude to the concentrations reported in this study. The high concentrations of steroid hormones in water systems in developing countries including South Africa, could be attributed to the fact that conventional wastewater treatment plants do not have many capabilities to properly remove organic contaminants such as steroid hormones. Some of the steroid hormones, specifically the hydrocortisone, it is currently administered to patients that are affected by covid-19 to aid in breathing complications.

### Reusability, regeneration and stability

Regeneration and reusability studies indicated that PET based UIO-66(Zr) could be reused until the fourth cycle without showing any major decrease in the percentage recoveries of the analytes from the sample. As shown in Fig. S8, after the fourth cycle, a recognizable decrease of percentage recoveries was noticed. The affinity loss of the for the steroid hormones was subjected to the repeatedly washing and elution. This has also resulted into losing the binding sites on the PET based UIO-66(Zr). Therefore, PET based UIO-66(Zr) was regarded as a stable PET based UIO-66(Zr) with good reusability properties. The stability of PET based UIO-66(Zr) in different organic solvents and water was investigated according to^56–58^. As shown in Fig. S9, PET based UIO-66 (Zr) was found to be chemically resistant to all the conditions as there was no change in the XRD patterns.

Furthermore, with investigation of the stability of the material in water, Leaching studies were conducted to find out how stable is the material in water. ICP-OES was used to find out if the Zr element does not leach into water during and after extraction. The concentrations of Zr detected was not detected (− 0.002 µg L^−1^) in water sample, which indicated that the material was stable in water and does not cause any notable secondary pollution.

### Adsorption mechanism of the analytes

Different adsorption mechanism processes among the MOF and the steroid hormones were examined. The dominant mechanism was the electrostatic interactions among the positive charges of UIO-66 and the negative charges on the steroid hormones. At a pH lower than the pKa value of steroid hormones, the hormones are found in a neutral form. Subsequently, at pH values greater than the pKa value, the steroid hormones are negatively charged. Hence the electrostatic interaction mechanism is pH dependant. The optimum pH of the presented extraction process was 8 and the pKa values of the steroid hormones are above around 8 of the point of zero charge. Therefore, electrostatic interactions among the steroid hormones and the positively charged UIO-66 had an important role in the adsorption process. Another adsorption mechanism which was observed was the hydrogen bond among the nitrogen atoms (hydrogen acceptors) of the UIO-66 and the hydrogen bond donors of the steroid hormones indicated by the blue line in Fig. S10. Finally, the π–π interactions (red line) between the aromatic compounds of the steroid hormones and rings on the UIO-66 occurred as one the interaction processes as shown in Fig. S9.

The FTIR of the PET based UIO-66(Zr) adsorbent after the adsorption of the analytes from water sample was carried out. This was conducted in order to study the differences between the pure and spent adsorbent material. The FTIR spectra (Fig. S11) is different from the FTIR spectra in Fig. S1 and the difference is brought by the presence of analytes in Fig. S11. Figure S11 indicated to have all the peaks which were observed in Fig. S1 of the PET based UIO-66(Zr) (red). However, Fig. S11 had additional peaks which were attributed to the presence of the analytes in the material. The first two peaks observed in Fig. S10 at around 3760 and 3394 cm^−1^ were for the O–H peak present in the estrone and 17β-estradiol and the PET based UIO-66(Zr). These peaks include the peak at around 2960 cm^−1^ and was attributed to the aliphatic carbons found in all the analytes (17β-estradiol, progesterone, hydrocortisone and estrone). These double-like peaks at around 2960 cm^−1^ were attributed to the C–H stretching in the alkenes and alkanes found in the analytes and the adsorbent. Another peak was observed at around 1700 cm^−1^ which was for the ketone functional group present in the hydrocortisone and progesterone analytes. The peak at around 1661 cm^−1^ was due to the aromatic ring of the analytes and the PET based UIO-66(Zr). These peaks were also caused by the C = O which was in the analytes and the adsorbent.

## Methods

### Chemicals and standards

All solvents and reagents were obtained commercially and were not modified before use. Methanol (MeOH, HPLC grade, 99.9%), zirconium tetrachloride (99.9%), formic acid (99%), and ethanol (98%) were purchased from Sigma-Aldrich (St Louis, MO, USA). The analytes of interest namely, progesterone (99%), hydrocortisone (98%), 17β-estradiol (99%) and estrone (99%) were purchased from Sigma-Aldrich. Table S1 shows the chemical structure, molar masses and Pk_a_ values, CAS number for the steroid hormones. Ultra-pure water (Direct- Q® 3UV-R purifier system) was used in all experiments. Stock solutions of the mixed steroid hormones (100 mg/L for each analyte) were prepared by dissolving suitable mass of the analytes in methanol and kept at 3 °C in amber glass bottles for the duration of the experiments. Working synthetic samples and standard solutions were prepared daily by diluting suitable volumes of stocks with methanol or ultrapure water or river water sample. It should be noted that methanol was used for the preparation of calibration standards used to calibrate the instruments.

### Instrumentations

Different characteristic properties of the synthesised material were investigated using transmission electron microscope (TEM JOEL JEM-2100, Tokyo, Japan), Perkin-Elmer Spectrum 100 Attenuated total reflection Fourier transform infrared (ATR-FTIR) (PerkinElmer, Waltham, MA, USA), Surface Area and Porosity Analyzer (ASAP2020 V3. 00H, Micromeritics Instrument Corporation, Norcross, USA) and scanning electron microscopy/ energy dispersive X-ray spectroscopy (SEM/EDS, TESCAN VEGA 3 XMU, LMH instrument, Brno, Czech Republic). The pH of the sample solutions was adjusted using an OHAUS starter 2100 pH meter (Pine Brook, NJ, USA). Sonication was done using an ultrasound bath (Bandelin Sonorex Digitec, Bandelin electronic GmbH&Co. KG,Berlin, Germany). Response: A TIBCO® Statistica™ package version 13 (StatSoft, Palo Alto, CA, USA) was used to process the optimization data (https://edelivery.tibco.com/storefront/eval/tibco-statistica-desktop/prod11850.html).

### Chromatographic system and conditions

An Agilent 1200 Infinity series HPLC equipped with a diode array detector (Agilent Technologies, Waldbronn, Germany) was employed for the quantification and analysis of the steroid hormones in the samples. The solvents for the mobile phase were composed of water and acetonitrile in the percentage of 75:25 (v/v). The mobile phase solvents were pumped into an Agilent Zorbax Eclipse Plus column (C18) (3.5 µm × 150 mm × 4.6 mm) (Agilent Newport, CA, USA). The injection volume of 10 µL and a flow rate of 1.0 mL/min was used for the entire analysis (isocratic). The column temperature was at 25 °C and the analysis of the steroid hormones was done at a wavelength of 242 nm for β-estradiol, 260 nm for hydrocortisone, 242 nm for estrone and 272 nm for progesterone. The solutions prepared were filtered using membrane filters with a pore size of 0.45 mm (25 mm filter) and transferred into sample vial.

The quantification of steroid hormones was also performed by LC–MS (Shimadzu, Japan) with two LC-20AD-XR binary pumps, a DGU-20A3 degasser, a SIL-20ACXR auto-sampler, a CTO-20AC column oven, FCV-20AH2 valve unit and one triple quadrupole mass spectrometer equipped with an electrospray ionization (ESI) interface. The column used was a Zorbax Eclipse Plus C18 column (3.5 µm × 150 mm × 4.6 mm) kept at 25 °C. The mobile phase was composed of water and acetonitrile mixture (55% water and 45% acetonitrile). A flowrate of 0.5 mL/min was used for the analysis (isocratic).

### Point of zero charge (pH_pzc_)

The pH at point of zero charge (pH_pzc_) of PET based UIO-66(Zr) was investigated using a altered method reported by^59,60^. The pH of the solutions in the range of 2–12, were prepared and adjusted by sodium hydroxide and diluted acetic acid solutions. Subsequently, 18 mg of sorbent was put into each sample bottle, sonicated for 48 h. The final pH values after sonication were measured. The pH_pzc_ was then obtained from a curve of (ΔpH) versus pH_i_.

### Synthesis of PET based UIO-66(Zr)

#### Preparation of the PET-derived ligand

Waste PET bottles were collected from the University of Johannesburg, Doornfontein campus. They were cut into small pieces, washed with deionised water and dried at 50 °C. For the synthesis of PET flakes from waste PET bottles, the method was adopted from the literature^61^. Briefly, 5 g of PET plastic, 100 mL of deionised water and 5 mL of ethylene glycol were put into a Teflon-lined reactor and heated at 210 °C for 12 h. The white product was washed with water and ethanol, then dried at 70 °C for 24 h.

#### Synthesis of UiO-66 using PET-derived ligand

About 2.12 g of zirconium tetrachloride and 1.36 g of PET-derived ligand were added into 100 mL of deionised water followed by 10 mL of formic acid and sonicated for 30 min. The mixture was transferred to a Teflon-lined autoclave and heated at 160 °C for 12 h. The white product was filtered and washed with ethanol, then dried at 50 °C overnight.

### Dispersive solid phase extraction procedure

**Figure 4:**
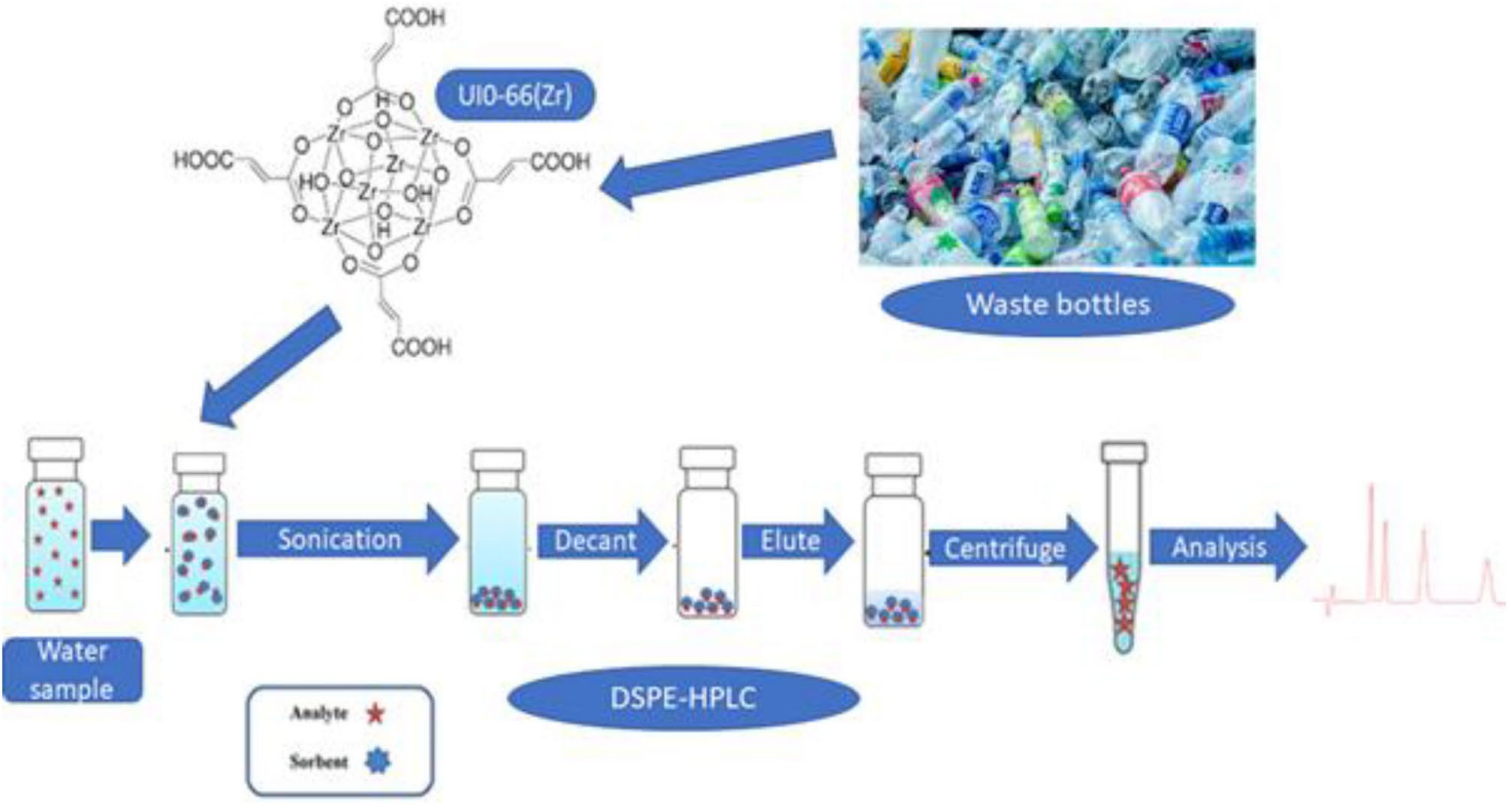
Dispersive solid phase extraction procedure.

The DPMSE procedure for extraction and preconcentration of hormones was carried out as follows: to describe the method briefly, 5 mL of model water samples (spiked with 100 µg/L of target analytes, pH = 4–9) was placed into a 10 mL sample bottle containing 15–20 mg of the adsorbent. The extraction and preconcentration of target analytes from the sample were achieved by sonicating the samples for 20–30 min. After extraction and preconcentration process, the adsorbent was separated from the aqueous solution via centrifugating at 3800 rpm for 10 min. The elution of the analytes was achieved by adding 500–100 µL of methanol and sonicated for 15 min. The eluent and the adsorbent were separated via centrifugation at 3800 rpm for 10 min. The eluent was filtered through 0.22 µm PVDF syringe filters before HPLC–DAD and UHPLC-MS/MS analysis. Figure 4 demonstrates how dispersive solid phase extraction was carried out.

#### Optimisation of DSPE procedure

The optimization of factors affecting the extraction and preconcentration of steroid hormones from water samples was achieved using Box-Behnken design (BBD). Four critical factors, including sample pH, eluent volume (EV) mass of adsorbent (MA) and extraction time (*ET*), were investigate at three levels (Table S2). This design was selected to assess the relationship between the investigated independent variables as wells as their combined interactive effects and analytical response values. This was achieved by designing a sequence of 27 experiments.

### Analytical method validation

#### Accuracy and precision

The accuracy of the developed method was evaluated using a spike recovery method. The river water samples were spiked at three levels (5, 10, and 100 ng/L) and the samples were extracted under optimum experimental conditions. The intraday and interday precisions were determined by analysing several replicates of spiked river water samples. For intraday, ten replicates were analysed in the same day while for interday precision, three replicates per day were measured for five consecutive working days.

#### Calibration curves, linearity, and matrix effect

Linearity was studied at seven different concentration levels ranging from 0.1 to 1200 µg L^−1^. The standard solutions were prepared by diluting stock solutions of each analyte with ultrapure water and the preconcentration of each standard was performed in triplicates. Matrix effects (%ME) are estimated by comparison of the slopes of calibration curves of each analyte made in ultrapure water and spiked river water samples. The equation below is used to express %ME.

2 $$MF = \frac{slope (matrix \,matched\,standard)}{{slope \left( {ultra\ pure\ water} \right)}}$$

3 $$\% ME = \left( {MF - 1} \right) \times 100$$

If matrix effects are found to be below − 50% or above + 50%, they are considered to have a strong effect. MEs are described as soft if they are within the range of − 20% % < MEs < 20%.

#### Limits of detection and quantification

The LOD and LOQ were calculated as: LOD = 3*sd*/*m* and LOQ = 10 sd/m, where ‘sd’ is the standard deviation of peak areas of the ten replicates of the lowest concentration level of the linear range and ‘m’ is the slope of each calibration curve of the target analytes.

### Sampling

Several river water samples were collected at different points from Rietspruit river (Sebokeng, South Africa) and Vaal River (Vaal, South Africa). Water samples at Rietspruit river were collected from upstream (before the wastewater treatment plant (WWTP); sample ID: RRU S1-4) and downstream (after WWTP; sample ID: RRD S5-7). Water samples at Vaal River were collected before (VRB S8 and S9) and after (VRA S10) Rietspruit river joins Vaal river, as well as downstream (VRD S11 and VRD S12). The physicochemical characteristics of water samples are summarized in Table S4. The river water samples were collected in 1000 mL amber glass bottles, kept in refrigerator at 4 °C for 5 days until analysis (the analysis of sample was done within one week). To eliminate particulates and suspended solids, water samples were filtered through 0.22 μm PVDF filter membranes before subjected to the optimised method.

### Quality assurance/quality control (QA/QC)

The Quality assurance/ Quality control of the developed method was done according to^21^. Blank samples were analysed using HPLC–DAD and LC–MS and there was no analyte of interests detected in the blank samples. These results gave guarantee that blank correction from all the examined water samples was not crucial for the developed method. When the water samples were analysed, standard solutions of the individual analytes at the concentration range 10–200 ng/L were prepared and analysed as QA/QC samples. Blank samples followed in the same way to real water samples and the above-stated QA/QC standard solutions were analysed with the HPLC–DAD after every tenth sample. When samples were lower than ten, the QA/QC procedure was followed every three samples.

### Reusability

Reusability and regeneration studies for PET based UIO-66(Zr) were done by performing a series of adsorption–desorption experiments followed by washing and drying of PET based UIO-66(Zr). Firstly, 19 mg of the sorbent was added into a glass bottle, then 5 mL of the synthetic sample was added into the glass bottle. The mixture was then sonicated for 30 min, then eluted with 750 µL of methanol. The eluent was analysed using HPLC–DAD. The used sorbent was washed with ultrapure water followed by ethanol and dried in an oven at 60 °C. The sorbent was then used for the following extraction and elution cycles.

## Conclusion

A quick, simple, and sensitive technique was designed and used for simultaneous preconcentration and extraction of different steroid hormones from river water samples. PET based UIO-66(Zr) was successfully synthesized and characterized then used for the solid phase microextraction. PET based UIO-66(Zr) showed to have great affinity towards the steroid hormones. The designed DSPE-HPLC–DAD method showed to be linear over a wide concentration range, precise, reproducible, and accurate. Furthermore, the designed method resulted into lower LODs and LOQs in comparison to other reported methods. PET based UIO-66(Zr) indicated to have high adsorption capacities towards the analytes in the range of 239–279 mg/g. The surface area of the was found to be 1311 m^2^ g^−1^ with a pore volume and an average pore size of 0.62 m^3^ g^−1^ and 3.76 nm, respectively. Hydrocortisone was detected in higher concentrations in Vaal River and Rietspruit river. Various adsorption mechanisms such as electrostatic interactions, hydrogen bonding, the π–π stackings were found to be leading most of the adsorption processes between the interaction of the steroid hormones and the PET based UIO-66(Zr). Finally, through regeneration studies, PET based UIO-66(Zr) was found to be reusable and regenerable for up to 4 cycles and leeching studies showed that the material does not leech in water.

## Supplementary Information

Supplementary Information.

## Data Availability

The raw data supporting the conclusions of this article will be made available by the authors, without undue reservation. The corresponding author (Philiswa N. Nomngongo) can be contacted if data is requested from this study.
