# Supplementary material for: From polyethylene waste bottles to UIO-66 (Zr) for preconcentration of steroid hormones from river water
Source: Sci Rep. 2023 Apr 26;13:6808. doi: 10.1038/s41598-023-34031-z (PMC10131548; doi:10.1038/s41598-023-34031-z)
Supplement: Supplementary file 1 — Supplementary Information. [file 41598_2023_34031_MOESM1_ESM.docx]

**From polyethylene waste bottles to UIO-66 (Zr) for preconcentration of steroid hormones from river water**

Shirley Kholofelo Selahle^1^, Azile Nqombolo^1,2,3^, Philiswa Nosizo Nomngongo^1,2*^

^1^ Department of Chemical Sciences, University of Johannesburg, Doornfontein Campus, P.O. Box 17011, Doornfontein, 2028, South Africa

^2^ Department of Science and Innovation-National Research Foundation South African Research Chair Initiative (DSI-NRF SARChI): Nanotechnology for Water, University of Johannesburg, Doornfontein, 2028, South Africa

^3^ Department of Pure and Applied Chemistry, University of Fort Hare, Alice, 5700, South Africa

***** Corresponding Author: Philiswa N. Nomngongo, p[nnomngongo@uj.ac.za](mailto:nnomngongo@uj.ac.za)

Supplementary data


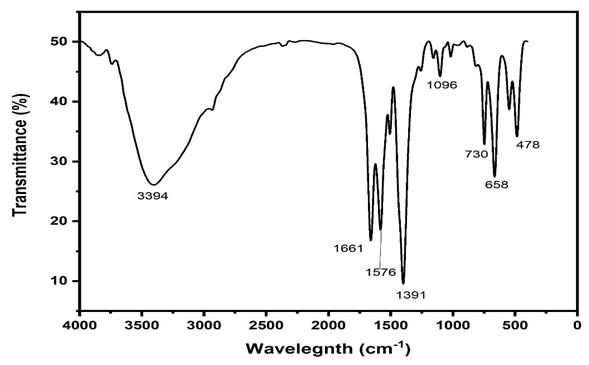


Figure S1: FTIR of PET based UIO-66(Zr)


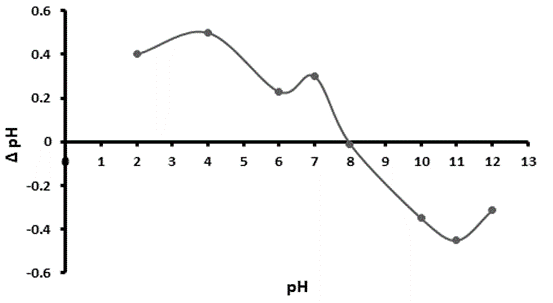


Figure S2: Point of zero charge OF PET based UIO-66(Zr)


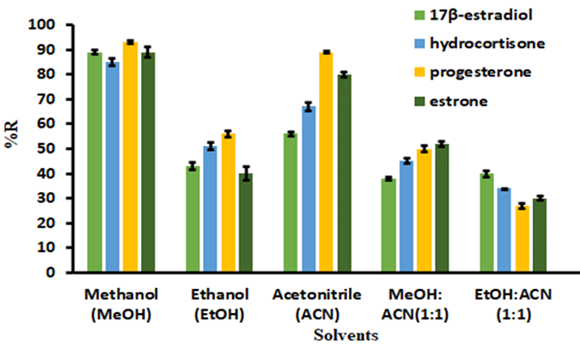


Figure S3: Choice of eluent solution


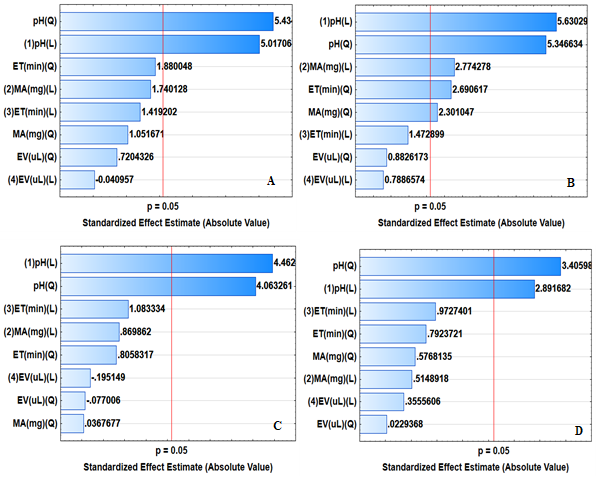


Figure S4: Pareto chart for standardized effects on variables for extraction and preconcentration of A) 17β-estradiol, B) estrone, C) Hydrocortisone and D) progesterone. MA (mg) =Mass of adsorbent, ET (min)= Extraction time, EV (uL)= Eluent volume.

(https://edelivery.tibco.com/storefront/eval/tibco-statistica-desktop/prod11850.html)


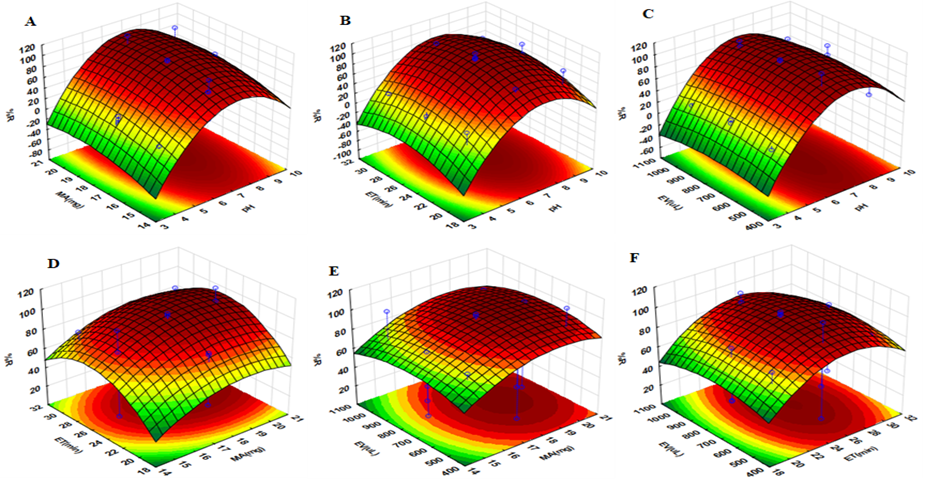


Figure S5: Three dimension response surface plots showing there interactions between: A) pH and mass of adsorbent (MA) while extraction time (ET) and eluent volume (EV) were fixed at 17.5 min and 750 µL; B) pH and ET while MA and EV were fixed at 25 mg and 750 µL; C) pH and EV while MA and ET were fixed at 25 mg and 17.5 min; D) ET and EV while pH amd MA were fixed at 6.5 and 25 mg; E) MA and ET while pH and EV were fixed at 6.5 and 750 µL; F) MA and EV while pH and ET were fixed at 6.5 mg and 17.5 min. A TIBCO® Statistica™ package version 13 (StatSoft, Palo Alto, CA, USA) (https://edelivery.tibco.com/storefront/eval/tibco-statistica-desktop/prod11850.html)


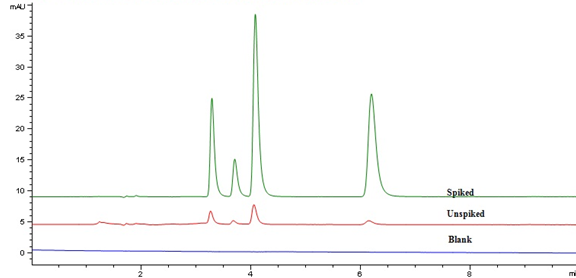


Figure S6: Chromatograms for spiked, unspiked and blank samples


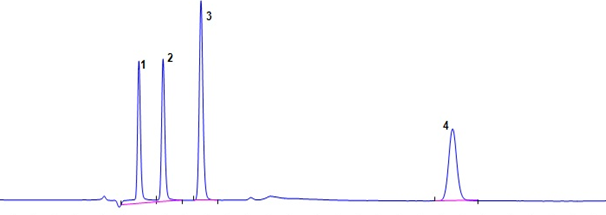


Figure S7: Chromatograms of Rietspruit river water sample showing the targeted analytes. (1) 17β-estradiol, (2) hydrocortisone, (3) estrone and (4) progesterone.


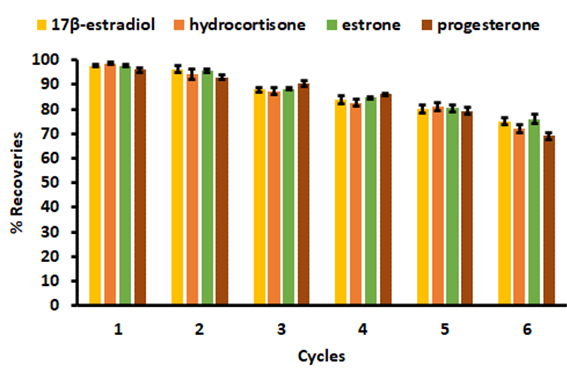


Figure S8: Regeneration and reusability.


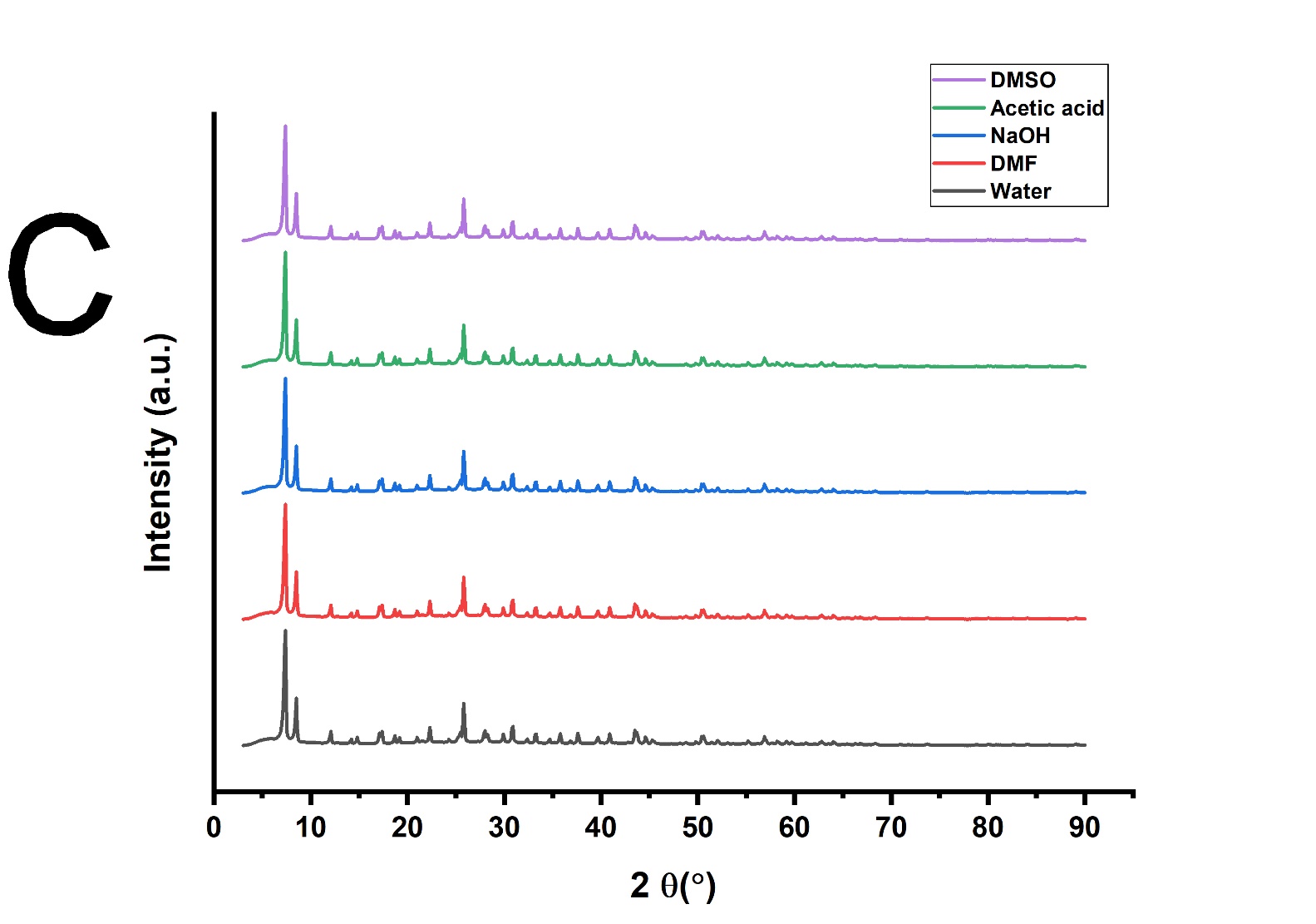


Figure S9: Stability of PET based UIO-66(Zr)


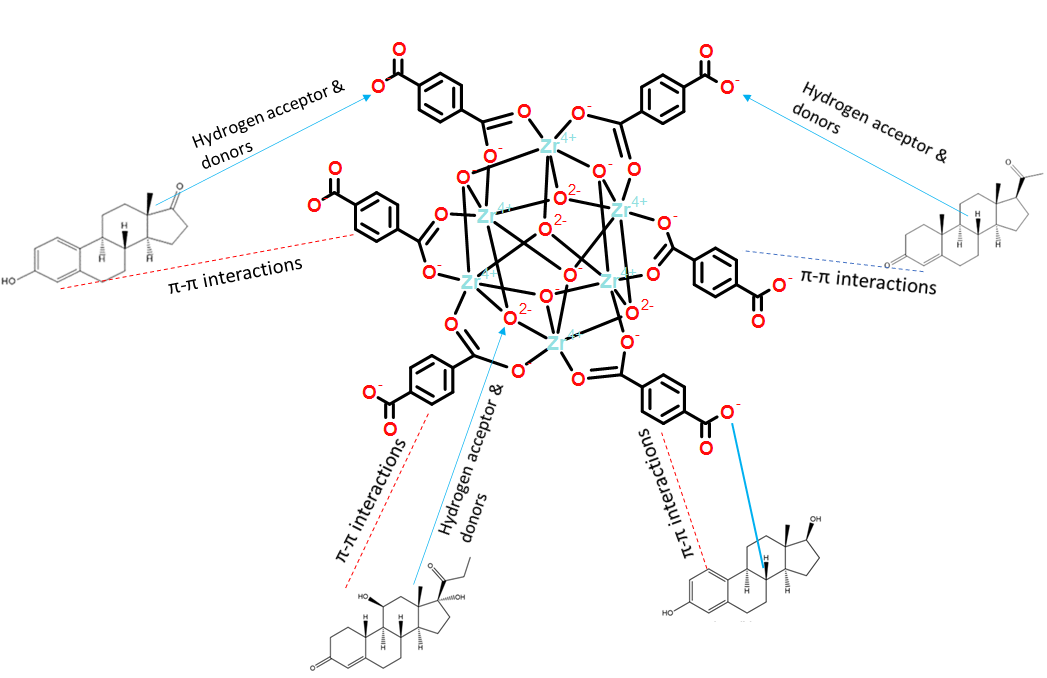


Figure S10: Interaction mechanisms


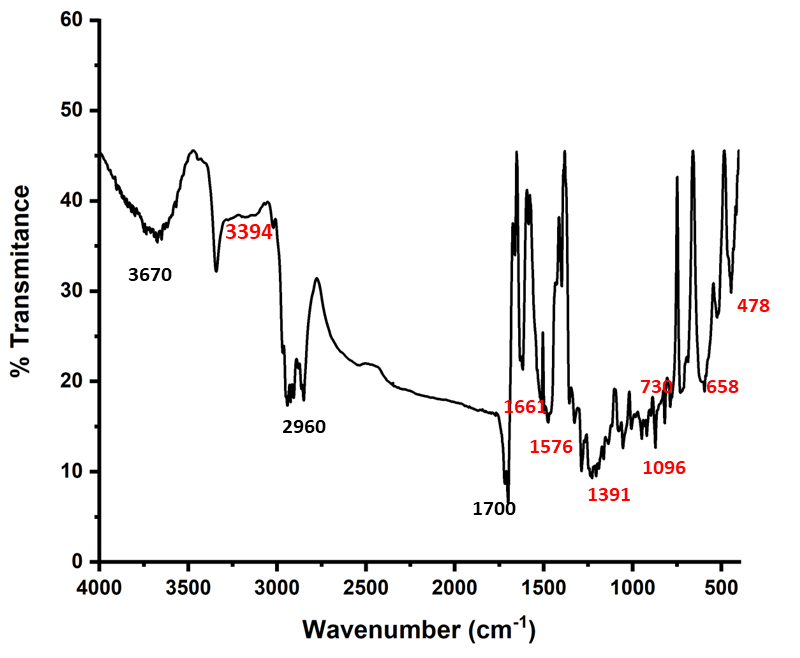


Figure S11: FTIR spectra of the PET based UIO-66(Zr) after adsorption of the analytes.

Table S1: BBD experimental results

| **Standards** | **4 Factors Box-Behnken design, 3v blocks, 27 runs** | | | | | **% Recoveries** | | |
| --- | --- | --- | --- | --- | --- | --- | --- | --- |
| **runs** | **Block** | **pH** | **MA** | **EV** | **ET** | **%hydro** | **% estradi** | **% estro** |
| 1 | 1 | 4 | 15 | 750 | 25 | 15.84 | 11.89 | 10.76 |
| 2 | 1 | 9 | 15 | 750 | 25 | 56.49 | 42.92 | 32.66 |
| 3 | 1 | 4 | 20 | 750 | 25 | 10.58 | 5.54 | 14.43 |
| 4 | 1 | 9 | 20 | 750 | 25 | 111.79 | 96.70 | 100.90 |
| 5 | 1 | 6.5 | 17.5 | 750 | 20 | 97.57 | 79.14 | 70.23 |
| 6 | 1 | 6.5 | 17.5 | 500 | 30 | 72.13 | 80.13 | 74.40 |
| 7 | 1 | 6.5 | 17.5 | 1000 | 20 | 55.72 | 50.40 | 60.58 |
| 8 | 1 | 6.5 | 17.5 | 1000 | 30 | 79.71 | 70.62 | 69.52 |
| 9 | 1 | 6.5 | 17.5 | 750 | 25 | 64.46 | 87.15 | 99.31 |
| 10 | 2 | 4 | 17.5 | 500 | 25 | 28.72 | 12.87 | 23.11 |
| 11 | 2 | 9 | 17.5 | 500 | 25 | 55.04 | 46.45 | 67.35 |
| 12 | 2 | 4 | 17.5 | 1000 | 25 | 15.75 | 17.02 | 31.19 |
| 13 | 2 | 9 | 17.5 | 1000 | 25 | 75.38 | 76.20 | 84.61 |
| 14 | 2 | 6.5 | 15 | 750 | 20 | 36.89 | 28.10 | 17.97 |
| 15 | 2 | 6.5 | 20 | 750 | 20 | 45.68 | 56.91 | 67.53 |
| 16 | 2 | 6.5 | 15 | 750 | 30 | 123.33 | 78.45 | 66.74 |
| 17 | 2 | 6.5 | 20 | 750 | 30 | 101.30 | 91.29 | 89.36 |
| 18 | 2 | 6.5 | 17.5 | 750 | 25 | 89.99 | 100.57 | 109.66 |
| 19 | 3 | 4 | 17.5 | 750 | 20 | 21.71 | 27.47 | 11.17 |
| 20 | 3 | 9 | 17.5 | 750 | 20 | 85.68 | 81.46 | 91.18 |
| 21 | 3 | 4 | 17.5 | 750 | 30 | 13.55 | 21.86 | 34.03 |
| 22 | 3 | 9 | 17.5 | 750 | 30 | 50.63 | 71.16 | 68.57 |
| 23 | 3 | 6.5 | 15 | 500 | 25 | 65.90 | 78.33 | 65.45 |
| 24 | 3 | 6.5 | 20 | 500 | 25 | 121.10 | 109.67 | 89.07 |
| 25 | 3 | 6.5 | 15 | 1000 | 25 | 99.90 | 99.89 | 99.22 |
| 26 | 3 | 6.5 | 20 | 1000 | 25 | 80.05 | 89.85 | 89.39 |
| 27 | 3 | 6.5 | 17.5 | 750 | 25 | 94.04 | 98.40 | 89.49 |

Table S2: The chemical structure, molar masses and Pk_a_ values, CAS number for the steroid hormones

| Steroid hormones | Structures | Molar masses (g mol^-1^) | LogKow | PK_a_ | CAS number |
| --- | --- | --- | --- | --- | --- |
| Estrone | 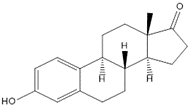 | 270.36 | 3.13 | 10.77 | 53-16-7 |
| Hydrocortisone | 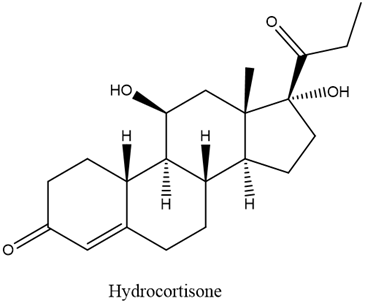 | 362.46 | 1.61 | 12.58 | 50-23-7 |
| 17β-estradiol | 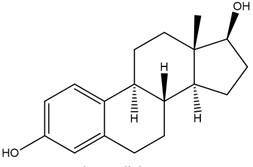 | 272.40 | 4.01 | 10.71 | 50-28-2 |
| Progesterone | 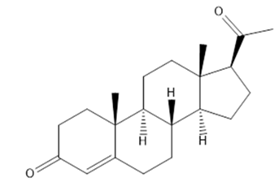 | 314.46 | 3.87 | 18.92 | 57-83-0 |

Table S3: Parameter and levels used in fractional factorial design

| parameters | Minimum | Central | Maximum |
| --- | --- | --- | --- |
| pH | 4 | 6.5 | 9 |
| Mass of sorbent (mg) | 15 | 17.5 | 20 |
| Eluent volume(µL) | 500 | 750 | 1000 |
| Extraction time (mins) | 20 | 25 | 30 |

Table S4: Physicochemical properties of the river water samples.

| Sample ID | pH | TDS (mg/L) | EC mS/m |
| --- | --- | --- | --- |
| RRU S1-4 | 8.3-9.6 | 290-300 | 0.57-0.60 |
| RRD S5-7 | 8.4-9.6 | 265-270 | 0.55-0.65 |
| VRB S8-9 | 8.6-8.8 | 270-280 | 0.54-0.55 |
| VRA S10 | 8.7-8.8 | 185-191 | 0.37-0.40 |
| VRD S11-12 | 8.5-9.0 | 190-195 | 0.37-0.41 |

TDS: Total dissolved solids, EC: Electrical conductivity

Table S5: Different adsorption capacities of PET based UIO-66(Zr) (mg/g) (average±std dev)

| Steroid hormones | Adsorption capacities (mg/g) |
| --- | --- |
| 17β-estradiol | 279±3 |
| Hydrocortisone | 256±4 |
| Estrone | 239±3 |
| Progesterone | 241±2 |

Table S6: Comparison of current work with other solid phase methods coupled with various analytical techniques for the detection of steroid hormones in aqueous matrices

| Analytes | matrix | Sorbent | Analytical techniques | Linearity | LOD | refs |
| --- | --- | --- | --- | --- | --- | --- |
| 17β-estradiol | wastewater | Fe_3_O_4_-Al_2_O_3_@CNFs | HPLC-DAD | 0.1-1000 µg/L | 25 ng/L | [21] |
| Glucocorticoids | Environmental water | Graphene oxide+ carbon nanotubes | HPLC-MS/MS | 0.05-1000 ng/L | 0.0075-0.16 ng/L | [22] |
| Estrone and 17β-estradiol | Environmental samples | MIL-101 (Cr) | UHPLC-MS/MS | 5- 50 000 ng/L | 0.95-23 ng/L | [53] |
| Estrogens | Surface water | HLB | GC-MS |  | 0.085-1.12 ng/L | [54] |
| Glucocorticoids | River water samples | (SDS-MWCNTs) | UHPLC-MS/MS | 0.2 -100 ng/mL | 0.019-0.098 ng/L | [22] |
| 17β-estradiol, hydrocortisone, estrone and progesterone | River water samples | PET based UIO-66(Zr) | HPLC-DAD | 0.06-1000 µg/L | 0.055-0.096 µg/L | This work |

n.d= not detected, HLB=Hydrophilic lipophilic balanced copolymer, SDS-MWCNTs= Sodium dodecyl sulfate-multi-walled carbon nanotubes

Table S7: Global concentrations of steroid hormones in water systems

| Countries | Steroid hormones | Sample matrice | Detected concentration (ng/L) | References |
| --- | --- | --- | --- | --- |
| Brazil | 17β-estradiol | River water | 14900 | [55] |
| South Africa | 17β-estradiol | Wastewater | 15-2000 | [56] |
| Poland | Estrone | Groundwater | 309 | [57] |
| China | Progesterone | River water | 8.22-66.2 | [58] |
| Brazil | Estrone, 17β-estradiol, progesterone, | Surface water | 0-5840 | [59] |
| South Africa | Progesterone, estrone, 17β-estradiol, estriol | Wastewater and river water | 0-7133 | [60] |
| South Africa | 17β-estradiol, estrone, hydrocortisone, progesterone | River water | 0-778 | This study |
